# Bacterial Profile and Antimicrobial Susceptibility Pattern of the Isolates from Stethoscope, Thermometer, and Inanimate Surfaces of Mizan-Tepi University Teaching Hospital, Southwest Ethiopia

**DOI:** 10.1155/2018/9824251

**Published:** 2018-06-27

**Authors:** Teshale Worku, Dejene Derseh, Abera Kumalo

**Affiliations:** ^1^Department of Biomedical Sciences, College of Health Sciences, Mizan-Tepi University, Mizan Aman, Ethiopia; ^2^Department of Medical Laboratory Science, College of Medicine and Health Sciences, Wolaita Sodo University, Sodo, Ethiopia

## Abstract

**Background:**

Nosocomial infections occur among patients during their stay in hospitals. The severity of infection depends on the characteristics of microorganisms with a high risk of being acquired when the environment is contaminated. Antibiotic-resistant bacteria are emerging rapidly around the globe creating a serious threat.

**Methods:**

A cross-sectional study was conducted from December 2016–February 2017 at Mizan-Tepi University Teaching Hospital, Southwest Ethiopia. Samples were collected from the equipment and hospital surfaces. The isolated bacteria were checked for susceptibility by the Kirby–Bauer disc diffusion method following the standards of CLSI 2014. Health professionals and sanitary team members were included in the study which assessed the disinfection practice of objects from which samples were taken. Data were analyzed using SPSS version 20.0.

**Results:**

A total of 201 swab samples were taken, and most bacteria were recovered from thermometer and floor consisting of 21.6% *S. aureus*, 19.3% CoNS, 15.9% *E. coli*, 14.8% *Klebsiella* species, 11.4% *P. aeruginosa*, 10.2% *Proteus* species, and 6.8% *Serratia* species. The most multidrug resistant organisms were *S. aureus* (79%), *Klebsiella* species (53.8%), CoNS (47%), and *Proteus* species (44.4%). Only 6.45% of health professionals disinfect their stethoscope consistently.

**Conclusion:**

*S. aureus*, CoNS, and *E. coli* were the predominant isolates. Most isolates showed highest susceptibility to ciprofloxacin and least to ampicillin and penicillin. There is no regular sanitation and disinfection of hospital equipment and surfaces.

## 1. Background

Nosocomial infections are those infections which are acquired during hospitalization [1]. The acquisition and severity of such infections depend on the characteristics of microorganisms and the rate of contamination of hospital environment [2]. Hospital surfaces and frequently used medical equipment are contaminated by a variety of pathogenic microorganisms [3–7]. Contamination of patient serving hospital environment increases the risk of healthcare-associated infections [6]. The hospital environment can be contaminated with bacterial pathogens such as *Staphylococcus aureus*, *Enterococcus*, *Streptococcus, Acinetobacter*, *Escherichia coli*, *Salmonella*, *Shigella*, *Klebsiella*, *Proteus*, and *Pseudomonas* spp. Environmental surfaces in healthcare centers act as a reservoir for bacteria and can as well serve as vectors of the bacterial pathogens [8–10]. The acquisition of nosocomial pathogens by a patient and the resultant development of infection depend on a multifaceted interplay between the environment, a pathogen, and a susceptible host [7]. Contamination of rooms of unaffected patients is due to viability of organisms shed by previous occupants. But it could also be due to horizontal transmission from healthcare workers, visitors, or asymptomatic carriers as well as dissemination of the organisms through air flow or other means [11, 12]. The incidence of hospital-associated infections due to emerging antimicrobial resistant organisms is also increasing leading to higher morbidity and mortality [13, 14]. Many ordinary surfaces such as side table/bench, floors, carpets, wall, and many other areas in the hospital environment may not be adequately decontaminated and can become reservoirs of pathogens [15, 16]. Commonly used disinfection techniques are sometimes incapable of eradicating fomites reservoirs of nosocomial pathogens such as methicillin-resistant *Staphylococcus aureus* [13, 17–20]. There is a paucity of data on the extent and nature of contamination and microbial profile of frequently used medical equipment and inanimate surfaces in healthcare facilities especially in the context of developing countries. Thus, this study was undertaken aiming at determining the profile of nosocomial bacteria isolated from the stethoscope, thermometer, and inanimate surfaces of Mizan-Tepi University Teaching Hospital for which the antimicrobial susceptibility test was performed.

## 2. Methods

### 2.1. Study Area, Study Design, and Participants

An institution-based cross-sectional study was conducted from 01 December 2016–30 February 2017 in Mizan-Tepi University Teaching Hospital which is located at Aman subtown and serves for approximately 1.5 million people living in Southwestern Ethiopia. Sample size was assumed as 250 considering the number of functional stethoscopes (42), thermometers (16), and surfaces of five wards (192): outpatient, emergency service, gynecology and obstetrics, pediatrics, and medical and surgical wards. Information about cleaning of hospital surfaces was obtained from governmentally organized sanitary team.

### 2.2. Data Collection and Laboratory Methods

Swab samples were taken from a total of 20 stethoscopes, 7 thermometers, which are in routine use of each ward, and 174 hospital surfaces which likely have contact with patients, visitors, and healthcare workers. Sterilized test tubes and cotton-tipped swabs moistened with normal saline were used to collect samples by swabbing from the diaphragm of the stethoscope, tip of the thermometer, and surfaces conveniently. Data to assess infection prevention practices of healthcare professionals were collected using a self-administered questionnaire.

### 2.3. Laboratory Methods

#### 2.3.1. Sample Processing

The swabbed samples were inoculated into MacConkey agar and mannitol salt agar. The inoculated agar plates were incubated at 37°C for 24–48 hours, and the growth was inspected to identify the bacteria. Presumptive identification of bacteria was done based on Gram reaction and colony characteristics. Confirmatory tests were done by enzymatic and biochemical properties of the organisms which were performed for pure colonies subcultured on nutrient agar from primary cultures for final identification of the isolates. Gram-negative rods were identified by performing a series of biochemical tests such as carbohydrate fermentation on triple sugar iron agar, Simon's citrate agar, and lysine iron agar. Indole production and motility was checked on the sulfide-indole-motility (SIM) medium. Urease production was inspected using urea agar base supplemented with 40% urea solution. Gram-positive cocci were identified based on their Gram reaction, catalase, and coagulase test results.

#### 2.3.2. Antimicrobial Susceptibility Testing

Antimicrobial susceptibility testing was performed for each bacterial isolates using Mueller–Hinton Agar (MHA) (Oxoid, England) by the Kirby–Bauer disc diffusion method following standard procedures. Consequently, three to five selected colonies of a pure culture of bacteria were taken and transferred to a tube containing 5 ml of sterile normal saline and mixed gently to form a homogeneous suspension until the turbidity of the suspension becomes adjusted to 0.5 McFarland. A sterile cotton swab was used to remove the excess suspension by gentle rotation of the swab against the surface of the tube. The swab was then used to distribute the bacteria evenly over the entire surface of MHA. The inoculated plates were left at room temperature to dry for 3 to 5 minutes, and a set of antibiotic discs were placed on the inoculated plates using sterile forceps and were allowed to stand for 30 minutes. The plates were incubated at 35°C for 16 to 18 hours, and the diameter of the zones of inhibition which were determined by the break points of antimicrobial discs were measured with a ruler and interpreted according to the standards of 2014 Clinical and Laboratory Standards Institute (CLSI). The antimicrobial discs used for susceptibility testing were amoxicillin (AML, 10 *μ*g), ampicillin (AMP, 10 *µ*g), ceftriaxone (CTR, 30 *µ*g), ciprofloxacin (CIP, 5 *μ*g), erythromycin (ERY, 15 *μ*g), gentamicin (GEN, 10 *µ*g), methicillin/cefoxitin (FOX, 30 *μ*g), and penicillin (PEN, 10 units).

#### 2.3.3. Data Analysis and Interpretation

Data obtained from laboratory results and questionnaire were entered and analyzed using SPSS version 20.0. Frequency distribution statistical analysis was used to compute the results.

## 3. Results

### 3.1. Nosocomial Bacterial Isolates

A total of 201 swab samples were taken from six wards; emergency, pediatrics, medical, adult, and pediatric outpatient department, gynecology and obstetrics, and the operating room of Mizan-Tepi University Teaching Hospital. The samples belong to frequently used medical equipment such as the stethoscope, thermometer, and inanimate surfaces of the hospital from which various bacterial pathogens were isolated as presented below (Tables 1 and 2). Accordingly, the samples were taken from 52 door handles, 27 floors, 34 bed surfaces, 21 walls, 20 stethoscopes, 7 thermometers, 27 table tops, and 13 window handles. The type of bacteria isolated from these objects consisted of *S. aureus* 19 (21.6%), CoNS 17 (19.3%), *E. coli* 14 (15.9%), *Klebsiella* 13 (14.9%), *P. aeruginosa* 10 (11.4%), *Proteus* 9 (10.2%), and *Serratia* (6/6.8%) giving a total of 88 (43.8%) isolates.

### 3.2. Antimicrobial Susceptibility Pattern

Most of the CoNS isolate (12/70.6%) showed susceptibility to gentamicin. Ten (58.8%) isolates showed susceptibility to ciprofloxacin, methicillin, and erythromycin as well. However, less number of the isolates became sensitive to ceftriaxone and penicillin, which is six (35.3%) and five (29.4%), respectively. Methicillin-resistant coagulase negative *Staphylococci* (MRCoNS) in this study are found to be ten (58.8%). Amoxicillin-sensitive CoNS in this study are found to be seven (41.2%) of the total 17 isolates. Regarding susceptibility of *S. aureus*, eleven (11/57.9%) showed susceptibility to ciprofloxacin and six (31.6%) became susceptible to gentamicin. Few isolates showed sensitivity to the remaining antimicrobials. Only three (15.8%) *S. aureus* was found to be sensitive to penicillin. Sensitivity to methicillin was shown by only five (26.3%) isolates implying the highest rate of methicillin resistance among the organism. Meaning, methicillin-resistant *Staphylococcus aureus* (MRSA) was found to be 73.7% which was resisted by fourteen isolates. Few isolates (26.3%) showed sensitivity to amoxicillin.

Susceptibility pattern of *E. coli* and other Gram-negative bacterial isolates is presented in Table 3 and indicates that eleven (78.6%) *E. coli* showed sensitivity to ciprofloxacin but only two (2/14.3%) became sensitivity to ampicillin out of fourteen isolated organisms. However, half of the isolates showed sensitivity to gentamicin. Most *Klebsiella* isolates (84.6%) were sensitive to ciprofloxacin followed by nine (69.2%) isolates which became sensitive to ceftriaxone. Unlike most *E. coli* isolates, sensitivity to ampicillin is shown by eight (61.5%) isolates of *Klebsiella* species. Similar numbers of *Klebsiella* species were also sensitive to gentamicin. *Proteus* species and *Pseudomonas aeruginosa* showed highest sensitivity to ciprofloxacin in which eight (89%) of both isolates became sensitive to the abovementioned drug. However, only three (30%) *P. aeruginosa* isolates were found to be sensitive to amoxicillin. *Proteus* species showed least sensitivity to ampicillin and ceftriaxone. Accordingly, four (44.4%) showed sensitivity to ampicillin and ceftriaxone, and two (22.2%) showed sensitivity to gentamicin. *Serratia* species showed highest sensitivity to ciprofloxacin and least sensitivity to gentamicin; that is, five (83.3%) were sensitive to ciprofloxacin, and three (50%) were found to be sensitive to gentamicin.

### 3.3. Multidrug Resistance Profile

According to the current definition of multidrug resistance, resistance to more than three classes of drugs was experienced by certain species of the isolated bacterial pathogens. Hence, in this study, eight (47%) CoNS were found to be multidrug resistant and of which two (11.8%) were methicillin resistant, as presented in Table 4. Unlike CoNS isolates, multidrug resistance was highly observed among most isolates (79%) of *S. aureus* of which 14 (73.7%) were MRSA. From Gram-negative bacterial isolates, *Klebsiella* species were found to be resistant to more drugs, as presented in Table 4. Hence, multidrug resistance was experienced among 7 (53.8%) species. *Proteus* and *Serratia* also showed multidrug resistance in which 4 (44.4%) *Proteus* and 2 (33.3%) *Serratia* became MDR. *P. aeruginosa* showed the least multidrug resistance which was experienced among 3 (30%) isolates. Four (21%) *S. aureus*, 2 (11.8%) CoNS, and 1 (7.1%) *E. coli* were found to be resistant to the tested drugs.

### 3.4. Disinfection Practice of Medical Equipment

Medical equipment such as stethoscopes and thermometers is classified as noncritical items contributing much to cross-contamination in healthcare settings. Yet the disinfection practice of this important equipment is ignored by most of the health professionals. In this study, of 79 health professionals working in the hospital, 62 were included in the study which assessed the disinfection practice of noncritical medical equipment, that is, the stethoscope and thermometer they use. Accordingly, 14 (22.5%) of them disinfect their stethoscope using alcohol while examining a patient, and the remaining 48 (77.5%) of them never disinfect it. Of those who disinfect their stethoscope, only 8 (12.9%) health professionals practice disinfection both before and after examining a patient. Of those who disinfect their stethoscope before and after examining a patient only 4 (6.45%) health professionals practice it consistently.

### 3.5. Disinfection Practice of Hospital Surfaces

Based on the information obtained from the sanitarian team leader, disinfection of hospital surfaces such as floor, wall, door handle, and table top of each ward is the responsibility of the sanitary team. They use bleach to clean and disinfect the floor twice a day. However, cleaning/disinfecting walls, door handles, and table tops is not the usual practice as explained by the sanitarian team leader. Disinfecting the bed surfaces and linens which are contaminated by patient discharges is left for nurses as their usual responsibility.

## 4. Discussion

In this study, the prominent bacterial isolate from 201 screened objects was *S. aureus* which accounted 19 (21.6%) followed by CoNS and *E. coli* which were found as 17 (19.3%) and 14 (16%), respectively. However, a study carried out in Jimma on 176 screened stethoscopes found that CoNS accounted for 103 (58.5%) followed by *S. aureus* (79, 44.8%) and *Klebsiella* species (12, 6.8%) [21]. The abovementioned study also found significant number of *Salmonella*, *Citrobacter*, and *Enterobacter* isolates but few number of *E. coli* isolates. The variation in number and type of nosocomial bacterial isolates between these two studies implies differences in environmental sanitation and hygiene practices of the two hospital settings. Another study conducted in Nigeria reported much number of *Staphylococci* and *Streptococci* which was 52 (59.1%) and 6 (6.8%), respectively, from 39 stethoscopes, 36 sphygmomanometers, and 13 clinical thermometers [22]. The existence of *Streptococci* as reported by the abovementioned study is somewhat different compared with the findings of previous studies done elsewhere and this study which was done here at Mizan-Tepi University Teaching Hospital. However, the predominated type of bacterial isolate which was *Staphylococci* is similar with the finding of this study [22]. In general, this study and studies carried out elsewhere reported *S. aureus* as the most commonly isolated bacterial species from various hospital equipment and surfaces [15]. In line with this, CoNS was reported as the frequently isolated organism from the stethoscope and clinical thermometer [21, 23].

As per the report of study conducted in Nigeria on bacterial contamination of stethoscopes used by health workers, of the tested antimicrobials, ampicillin and erythromycin were resisted by all the isolates of *S. aureus*, *P. aeruginosa*, and *E. coli* [22]. This differs with the finding of the current study, even though 71.4% *E. coli* and 40% *P. aeruginosa* were found to be resistant to ampicillin and 73.7% *S. aureus* which resisted erythromycin. However, all isolates of *S. aureus* and most isolates of *P. aeruginosa* and *E. coli* became sensitive to ciprofloxacin which is also in contrary to the finding of this study in which certain isolates were found to be resistant to the abovementioned antimicrobials. Another study conducted in Iran on bacterial contamination and resistance to commonly used antimicrobials of healthcare workers' mobile phones in teaching hospital of Kerman indicated that 79% of the bacterial isolates were sensitive to both tested gentamicin and amoxicillin [24]. This is in contrary to the finding of the current study in which most bacterial isolates became resistant to the abovementioned antimicrobials. According to the finding of the study done in Jimma among bacterial isolates of stethoscopes, higher resistance of *P. aeruginosa* and CoNS to penicillin was reported as 75.9% and 87%, respectively [21]. But in this study, penicillin-resistant CoNS were documented as 64.7% which is lower than the finding of the former study. Methicillin resistance which was reported as 26.6% among *S. aureus* and 30.1% to that of CoNS [21] differs with the current finding which found only 12.5% CoNS and higher number of *S. aureus* (73.7%) which was documented as methicillin resistant. In the current study, multidrug resistance was observed among certain bacterial isolates. This was common among both Gram-positive and Gram-negative bacterial isolates. Even though multidrug resistance was observed among species of both groups of bacteria, *S. aureus* and *Klebsiella* showed highest resistance to multiple classes of drugs from Gram-positive and Gram-negative isolates respectively. Consequently, 15 (79%) *S. aureus* and 7 (53.8%) *Klebsiella* species were found to be multidrug resistant. Multidrug resistance observed by the remaining species of Gram-positive and Gram-negative bacteria as presented in Table 4 was 8 (47%) for CoNS and 4 (44.4%) for *Proteus* species which was considered significant. Generally, multidrug resistance among Gram-positive bacteria and that of Gram-negatives was 63% and 38%, respectively.

In the study conducted in Zaria, Nigeria found different finding to the current study in which only 11.1% *P. aeruginosa* and 7% *E. coli* were multidrug resistant [19]. But in the current study, multidrug-resistant *P. aeruginosa* and *E. coli* were documented as 30% and 28.6%, respectively. However, multidrug-resistant *S. aureus* which was reported as 31.3% in the previous study [19] is higher than that of the current one in which 22.1% of the organism was found to be multidrug resistant.

In this study, only 22.5% health professionals disinfect their stethoscope before and after examining each patient. This is inconsistent with the finding reported from the study carried out in Jimma in which only 13.6% of the operating room and 4.3% of the surgical ward attendants disinfect their stethoscope regularly before and after examining each patient [21]. As reported by the sanitary team, disinfection of the floor which is done once a day and immediately following patient body discharge is consistent with the finding reported from India. The study also reported that walls of OR and ICU were cleaned and disinfected once every day, and walls of each wards were washed once a week by mixing soda and soft soap in a 1 : 3 ratio [25]. However, in this study, the sanitary team reported that they have no idea of regular cleaning and disinfection of walls and ceilings except accidental conditions where there may be visible contamination. In addition to that our study showed that the high contamination rate of stethoscopes with potential pathogens may cause different types of diseases. Therefore, strict devotion to stethoscope disinfection and also to infection prevention procedures for possible other medical equipments may minimize nosocomial infections and ensure improved patient safety in hospital environment. Government body as well as colleges or university should strengthen awareness campaigns to all health professionals or health students' curriculum on improved hygienic practices so as to reduce the rate of infections and spread of bacterial pathogens that may lead to hospital-acquired infections.

## 5. Conclusion

*S. aureus*, CoNS, and *E. coli* were the predominant isolates. Most isolates showed highest susceptibility to ciprofloxacin and least to ampicillin and penicillin. There is no regular sanitation and disinfection of hospital equipment and surfaces. Therefore, continuous discussion and follow-up should be needed by stake holders to develop a habit of routine sanitation and disinfection of hospital surfaces and equipment.

## Figures and Tables

**Table 1:** Type and number of materials or surfaces screened for nosocomial bacteria at Mizan-Tepi University Teaching Hospital, 2017.

Ward	Number of objects sampled	Number of culture-positive samples	Type (number of organisms isolated)
Emergency	Door handle (10)	2	*S. aureus* (1), CoNS (1)
	Floor (4)	3^*∗*^	*S. aureus* (2), CoNS (1), *Klebsiella* spp. (1)
	Bed (3)	2	*E. coli* (1), *Proteus* spp. (1)
	Wall (3)	2	*Klebsiella* spp. (1),
			*P. aeruginosa* (1)
	Stethoscope (3)	1	*Klebsiella* spp. (1)
	Thermometer (2)	1	CoNS (1)
	Examination table (5)	2^*∗*^	*Klebsiella* spp. (1), *Serratia* spp. (1)
Pediatrics	Door handle (8)	3	*S. aureus* (2), *P. aeruginosa* (1)
	Floor (4)	4	*S. aureus* (3), CoNS (1)
	Bed (4)	3	*S. aureus* (2), CoNS (1)
	Wall (3)	2^*∗*^	*E. coli* (1), *Klebsiella* spp. (1), *Proteus* (1)
	Stethoscope (2)	1	CoNS (1)
	Thermometer (1)	1	CoNS (1)
	Bed-side table (4)	1	CoNS (1)
Medical	Door handle (6)	1	*Klebsiella* spp. (1)
	Floor (3)	2^*∗*^	*CoNS* (2), *P. aeruginosa* (1)
	Bed (7)	3^*∗*^	*S. aureus* (3), *CoNS* (1)
	Wall (3)	1^*∗*^	*E. coli* (1), *P. aeruginosa* (1)
	Stethoscope (2)	-	-
	Thermometer (1)	1^*∗*^	*S. aureus* (1), CoNS (1)
	Bed-side table (5)	1	*S. aureus* (1)
Adult and pediatrics OPD	Door handle (16)	2	CoNS (1), *P. aeruginosa* (1)
	Floor (8)	2	CoNS (1), *Serratia* spp. (1)
	Bed (8)	1	CoNS (1)
	Wall (6)	1	*Proteus* spp. (1)
	Stethoscope (8)	2	*E. coli* (1), *Serratia* spp. (1)
	Thermometer (3)	1^*∗*^	*Klebsiella* spp. (1), *Proteus* spp. (1)
	Examination table (5)	3	*E. coli* (1), *P. aeruginosa* (1), *Serratia* spp. (1)
	Window handle (7)	2	*E. coli* (2), *Klebsiella* spp. (1)
Gynecology and obstetrics	Door handle (7)	4	*S. aureus* (1), *Proteus* (2), *P. aeruginosa* (1)
	Floor (4)	1^*∗*^	*S. aureus* (1), *P. aeruginosa* (1)
	Bed (6)	2	*E. coli* (1), *Serratia* spp. (1)
	Wall (3)	1	*P. aeruginosa* (1)
	Stethoscope (3)	1	*Klebsiella* spp. (1)
	Bed-side table (5)	3	*E. coli* (2), *Klebsiella* spp. (1)
	Window handle (2)	1	*Serratia* spp. (1)
Operating room (OR)	Door handle (5)	2	*E. coli* (1)
	Floor (4)	2^*∗*^	*Klebsiella* spp. (1), *S. aureus* (2), *Proteus* species (1)
	Bed (6)	3	CoNS (2), *E. coli* (1)
	Wall (3)	2	*E. coli* (1), *Klebsiella* spp. (1)
	Stethoscope (2)	-	-
	Bed-side table (3)	2^*∗*^	*Proteus* spp. (2), *P. aeruginosa* (1)
	Window handle (4)	1	*E. coli* (1), *Klebsiella* spp. (1)

^*∗*^Mixed growth of bacteria; -, no growth of bacteria; OPD, outpatient department.

**Table 2:** Total number of screened equipment and surfaces and organisms detected at Mizan-Tepi University Teaching Hospital, 2017.

Type and number of screened object	Type of organism detected							Total
	CoNS	*E. coli*	*Klebsiella*	*Proteus*	*P. aeruginosa*	*Serratia*	*S. aureus*	
Door handle (52)	2	1	1	2	3	-	4	13
Floor (27)	5	-	2	1	2	1	8	19
Bed surface (34)	5	3	-	1	-	1	5	15
Wall (21)	-	3	3	2	3	-	-	11
Stethoscope (20)	1	1	2	-	-	1	-	5
Thermometer (7)	3	-	1	1	-	-	1	6
Table top (27)	1	3	2	2	2	2	1	13
Window handle (13)	-	3	2	-	-	1	-	6
*Total* = *201*	**17 (19.3)**	**14 (15.9)**	**13 (14.8)**	**9 (10.2)**	**10 (11.4)**	**6 (6.8)**	**19 (21.6)**	**88**

-, no detection of specified organism in that particular object.

**Table 3:** Antimicrobial susceptibility pattern of nosocomial bacteria isolated at Mizan-Tepi University Teaching Hospital, 2017.

Bacterial isolate	Antimicrobials and their effect on bacterial isolates																							
	AMP			AMX			CTR			CIP			ERY			FOX			GEN			PEN		
	S	I	R	S	I	R	S	I	R	S	I	R	S	I	R	S	I	R	S	I	R	S	I	R
CoNS	-	-	-	7	3	7	7	0	10	11	6	0	10	1	6	11	0	6	13	2	2	5	1	11
*E. coli*	2	2	10	6	2	6	6	2	6	11	2	1	-	-	-	-	-	-	7	3	4	-	-	-
*Klebsiella* spp.	8	0	5	6	4	3	9	2	2	11	1	1	-	-	-	-	-	-	8	2	3	-	-	-
*Proteus* spp.	4	1	4	6	1	2	4	2	3	8	1	0	-	-	-	-	-	-	2	4	3	-	-	-
*P. aeruginosa*	5	1	4	3	0	7	6	2	2	9	1	0	-	-	-	-	-	-	6	1	3	-	-	-
*Serratia* spp.	4	1	1	3	0	3	4	1	1	5	1	0	-	-	-	-	-	-	3	1	2	-	-	-
*S. aureus*	-	-	-	5	2	12	2	0	17	11	2	6	3	2	14	5	0	14	6	0	13	3	0	16

-, susceptibility test not done; AMP, ampicillin; AMX, amoxicillin; CTR, ceftriaxone; CIP, ciprofloxacin; ERY, erythromycin; FOX, cefoxitin; GEN, gentamicin; PEN, penicillin.

**Table 4:** Multidrug resistance profile of nosocomial bacteria at Mizan-Tepi University Teaching Hospital, 2017.

Bacterial isolate	Number of MDR organism	Antimicrobials resisted by most isolates
CoNS	8/17 (47%)	AMX, ERY, PEN
*E. coli*	4/14 (28.6%)	AMP, AMX, CTR, GEN,
*Klebsiella*	7/13 (53.8%)	AMP, AMX, GEN
*Proteus*	4/9 (44.4%)	AMP, AMX, GEN
*P. aeruginosa*	3/10 (30%)	AMP, AMX, CTR, GEN
*Serratia*	2/6 (33.3%)	AMP, AMX, GEN
*S. aureus*	15/19 (79%)	AMX, CTR, ERY, FOX, GEN, PEN

MDR = multidrug resistant.

## Data Availability

All relevant data are within the article, but any additional data required are available from the corresponding author upon request.
